# GPCR-like Protein ZmCOLD1 Regulate Plant Height in an ABA Manner

**DOI:** 10.3390/ijms252111755

**Published:** 2024-11-01

**Authors:** Xinyuan Zhang, Zhen Zhang, Hui Peng, Zimeng Wang, Heng Li, Yongqi Duan, Shuo Chen, Xidong Chen, Jinlei Dong, Weina Si, Longjiang Gu

**Affiliations:** National Engineering Laboratory of Crop Stress Resistance Breeding, School of Life Sciences, Anhui Agricultural University, Hefei 230036, China; zxy1909241497@163.com (X.Z.); 18156362997@163.com (Z.Z.); phui0519@163.com (H.P.); wzmhanchang@163.com (Z.W.); lh0829100399@163.com (H.L.); dyq1690145665@163.com (Y.D.); chenshuoz01@163.com (S.C.); cxd20011201@163.com (X.C.); m18555400360@163.com (J.D.)

**Keywords:** maize, plant height, GPCR-like protein, COLD1

## Abstract

G protein-coupled receptors (GPCRs) are sensors for the G protein complex to sense changes in environmental factors and molecular switches for G protein complex signal transduction. In this study, the homologous gene of GPCR-like proteins was identified from maize and named as ZmCOLD1. Subcellular analysis showed that the ZmCOLD1 protein is localized to the cell membrane and endoplasmic reticulum. A CRISPR/Cas9 knock-out line of *ZmCOLD1* was further created and its plant height was significantly lower than the wild-type maize at both the seedling and adult stages. Histological analysis showed that the increased cell number but significantly smaller cell size may result in dwarfing of *zmcold1*, indicating that the *ZmCOLD1* gene could regulate plant height development by affecting the cell division process. Additionally, ZmCOLD1 was verified to interact with the maize Gα subunit, ZmCT2, though the central hydrophilic loop domain by in vivo and in vitro methods. Abscisic acid (ABA) sensitivity analysis by seed germination assays exhibited that *zmcold1* were hypersensitive to ABA, indicating its important roles in ABA signaling. Finally, transcriptome analysis was performed to investigate the transcriptional change in *zmcold1* mutant. Overall, ZmCOLD1 functions as a GPCR-like protein and an important regulator to plant height.

## 1. Introduction

Heterotrimeric G protein complexes are essential for cells to sense environmental signals and to transduce and cascade them within the cell [1,2,3,4]. The heterotrimeric G protein complex is highly conserved in eukaryotes and consists of three main subunits, Gα, Gβ, and Gγ. When cells are stimulated by external signals, Gα binds guanosine 5′-triphosphate (GTP) and activates, releasing GβGγ dimers [5]. The activated Gα-GTP and the free GβGγ dimer bind to a large number of downstream effector genes and promote signal transduction, respectively. When signal transduction is completed, Gα-GTP is hydrolyzed to re-form Gα-GDP, which then binds the free GβGγ dimer to re-form a heterotrimeric G protein complex that can be re-stimulated by external signals [6,7]. In metazoan systems, the transition of activated and inactive state of Gα was found to be regulated by G protein-coupled receptors (GPCRs). GPCRs are characterized by mediating GDP release and GTP binding. The canonical GPCRs play as guanine nucleotide exchange factors (GEFs) and a classic GPCR protein sequence consists of an extracellular N domain, seven transmembrane domains, and a cytoplasmic C-terminated tail [8]. Some plant GPCR-like proteins contain the ABA_GPCR domain [9]. GPCRs binding to ligands leads to a change in Gα conformation, which in turn changes from an inactivated state (Gα-GDP) to an activated state (Gα-GTP), initiating G protein signaling [6]. Additionally, the transition of activated and inactive state of Gα was found to be regulated by regulators of G protein signaling (RGS), which has the activity of GTPase-accelerating protein (GAP) activity to promote GTP hydrolysis [10,11,12].

It follows that the perception of external signals by GPCRs is a critical first step in the G protein signaling pathway, and GPCRs play important roles in all aspects of the lifecycles. In contrast, some reports showed that Gα proteins could self-activated without a GPCR [13,14,15]. Some GPCR-like proteins were also identified in plant kingdoms and showed differences with canonical GPCRs in mammals, without GEF activity [13]. *Arabidopsis* GCR1 is the first GPCR-like protein identified in the plant genome [8], which contains seven transmembrane structural domains with high sequence similarity to the non-plant GPCRs. In the presence of ABA, root elongation of T-DNA-inserted *gcr1* mutants was significantly inhibited and the expression of ABA-related genes was significantly increased [8]. In addition, *Arabidopsis* G protein-coupled receptor 1 (GTG1) and G protein-coupled receptor 2 (GTG2) proteins, which have high sequence similarity to human GPR89 proteins, are able to bind Gα proteins and possess GTPase activity and ABA binding ability [2]. *Arabidopsis gtg1gtg2* mutants exhibit reduced ABA sensitivity, confirming that GTG1 and GTG2 are novel ABA receptor-type G protein-coupled receptors localized at the cell membrane [16]. The homologs of GTG1 and GTG2 in rice, OsCOLD1, localized in endoplasmic reticulum (ER) and plasma membrane, can bind to Gα proteins, activate GTPase activity of Gα proteins, and open Ca^2+^ channels to rapidly sense environmental temperature changes and enhance cold tolerance in rice [10]. Overexpression of the *TaCOLD1* gene, the homolog of GTG1 and GTG2 in wheat, resulted in a significant reduction in the height of the transgenic plants. It was further confirmed using in vivo and in vitro experiments that the TaCOLD1 protein binds to the carbon end of TaGα-7A through the intracellular hydrophilic structural domain, and the C-terminus of TaGα-7A can bind to the TaDEP1 (wheat Gγ subunit) protein [17].

Maize is one of the most important staple crops worldwide. Plant height is a critical agronomic trait associated tightly with the maize yield and overall productivity [18]. In this study, we identified a maize gene, *ZmCOLD1*, which has been characterized as the orthologous gene of *GTG1*, *GTG2*, and *COLD1* [19]. With construction of a *zmcold1* mutant, the biological function of *ZmCOLD1* in regulation of plant height was characterized. The physiological mechanism underlying the phenotype of *zmcold1* was also comprehensively determined. Furthermore, the interaction between ZmCOLD1 and maize Gα subunit and the necessary domain underlying their interaction was confirmed by in vivo and vitro methods. The ABA sensitivity of *zmcold1* mutant was further assessed by seed germination assays. Finally, transcriptome analysis was conducted to decipher the transcriptional regulation of ZmCODL1. Overall, this study is of great theoretical significance to identify the key genes involved in the G protein signal transduction pathway in maize, which would benefit maize breeding in future.

## 2. Results

### 2.1. ZmCOLD1 Gene Bioinformatics Analysis

In the present study, the protein encoded by *Zm00001d026239* showed the highest sequence similarity with the GTG-type G protein-coupled receptors, GTG1 and GTG2 in *Arabidopsis* and OsCOLD1 in *O. sativa*, according to blast search. *Zm00001d026239* was therefore named as *ZmCOLD1*. Subsequently, a Neighbor-Joining phylogenetic tree was constructed with other GTG-type G protein-coupled receptor homologous from sorghum (Sb006G203300.1), short-stalked grass (Bd5g20597.1), tomato (Sl07g045330), alfalfa (Medtr5g005440.1), soybean (Gm12G014800.1 and Gm11G109900.1), southern cypress (Sm235006), and Rhizobium (Cre16.g674739.t1.1) plants, as well as humans (HsGPR89A) and nematodes (CeC11H1.2, CeY75B8A.16) (Figure S1).

**Figure 1:**
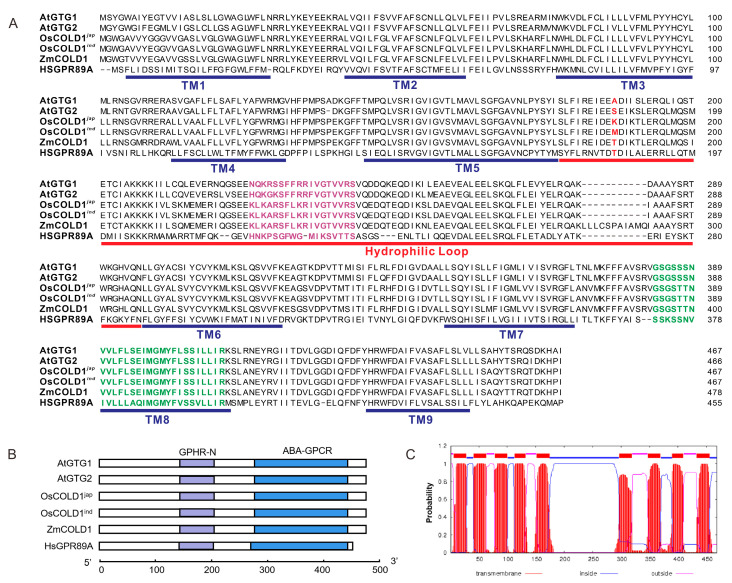
Homologous and structural analysis of ZmCOLD1 in maize. (**A**) Sequence alignments of ZmCOLD1 and homologous proteins. Purple sequence represents RAS-GTPase active region and green sequence represents ATP-/GTP binding region. The red letters in red is the putative conserved amino acid. (**B**) Conserved domain composition of ZmCOLD1 and homologous proteins. (**C**) Topology prediction for ZmCOLD1 using a transmembrane domain hidden Markov model (TMHMM version 2.0).

To further analyze the sequence characteristics of GTG-type G protein-coupled receptors, the amino acid sequence of ZmCOLD1 was aligned with *Arabidopsis* AtGTG1/AtGTG2, and rice OsCOLD1 protein sequences (Figure 1A). The GTG-type G protein-coupled receptor from humans, GPR89A, was used as a control. Results revealed that ZmCOLD1 harbored nine predicted transmembrane (TM) domains, similar to the other four proteins. There was a conserved hydrophilic loop between TM5 and TM6. In addition, a RAS-GTPase active region and an ATP-/GTP binding region were presented in all surveyed proteins. In contrast, the sequences of the two regions in ZmCOLD1 showed higher similarity with those in OsCOLD1. Furthermore, domain composition analysis showed that these proteins contain two typical structural domains, namely GPHR-N and ABA-GPCR (Figure 1B). Prediction of subcellular localization with a transmembrane domain hidden Markov model suggested that ZmCOLD1 proteins were typical transmembrane proteins with nine transmembrane domains (Figure 1C).

### 2.2. Tissue-Specific Expression Pattern Analysis of ZmCOLD1

**Figure 2:**
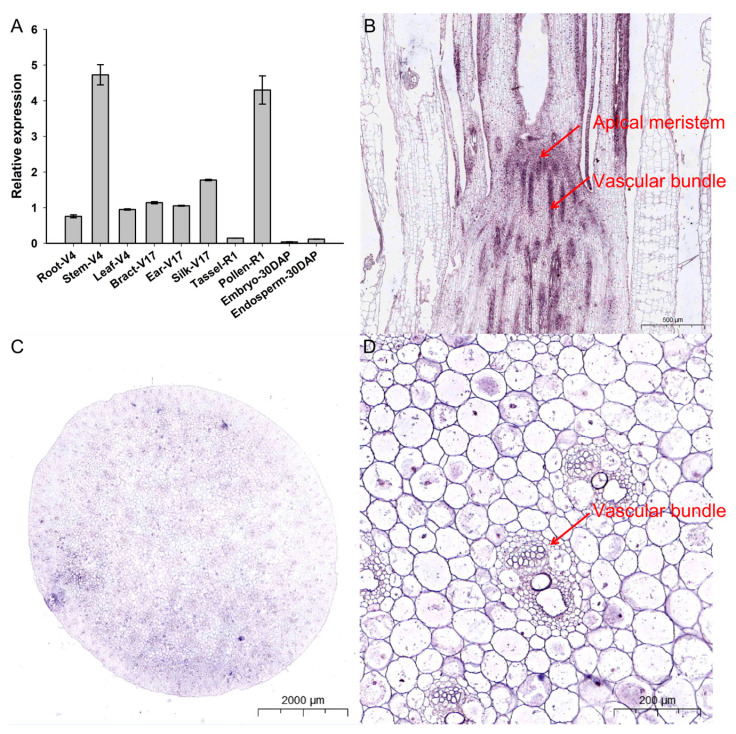
Tissue expression pattern analysis and in situ hybridization of *ZmCOLD1* (**A**) Tissue expression pattern analysis of *ZmCOLD1*; (**B**–**D**) RNA in situ hybridization of *ZmCOLD1*. (**B**) Longitudinal cut of the stem; (**C**) cross-section of the stem; (**D**) enlarged region of (**C**).

The tissue-specific expression profiles of *ZmCOLD1* were identified in different tissues of maize, including root, stem, and leaves of four-leaf stage, bract, ear, and filament of 17-leaf stage, R1 male panicles, R1 pollen, embryos, and endosperms at 30 days after pollination (Figure 2A). *ZmCOLD1* was expressed in root, stem, leave, bract, ear, filament, and pollen, with the highest expression in maize stem and pollen. This suggests that *ZmCOLD1* may be related to plant development, stem elongation, and pollen development during plant development. In order to further determine the expression distribution of *ZmCOLD1* gene in tissues, the expression of *ZmCOLD1* in stem (V6) tissue sections was detected by in situ hybridization technology. The transcript of *ZmCOLD1* can be detected in apical meristem (Figure 2B) and stem tissues at V6 stage, which were highly expressed in vascular bundle (Figure 2C,D).

**Figure 3:**
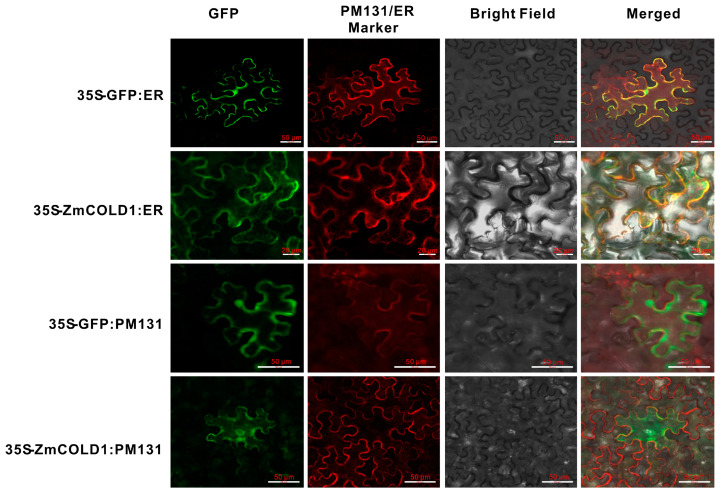
Subcellular localizations of ZmCOLD1 in tobacco leaves. Scale bar, 20 μm. PM131—cell membrane localization marker; ER—localization marker for endoplasmic reticulum.

To further validate the predicted results of subcellular localization of ZmCOLD1 protein, we transiently co-expressed ZmCOLD1-GFP fusion protein and the endoplasmic reticulum (ER)-localized marker gene in tobacco. The cellular green fluorescent signal of ZmCOLD1-GFP fusion protein was clearly visible and co-localized with the red fluorescent signal of the endoplasmic reticulum (ER)-localized marker gene. Moreover, we co-expressed ZmCOLD1-GFP with the membrane localization signal (PM131) in tobacco transiently and found that the ZmCOLD1 protein overlapped with the red fluorescent signal of PM131, indicating that ZmCOLD1 was expressed on the cell membrane as well as the endoplasmic reticulum (Figure 3).

### 2.3. Knock-Out Zmcold1 Caused a Dwarf Phenotype

**Figure 4:**
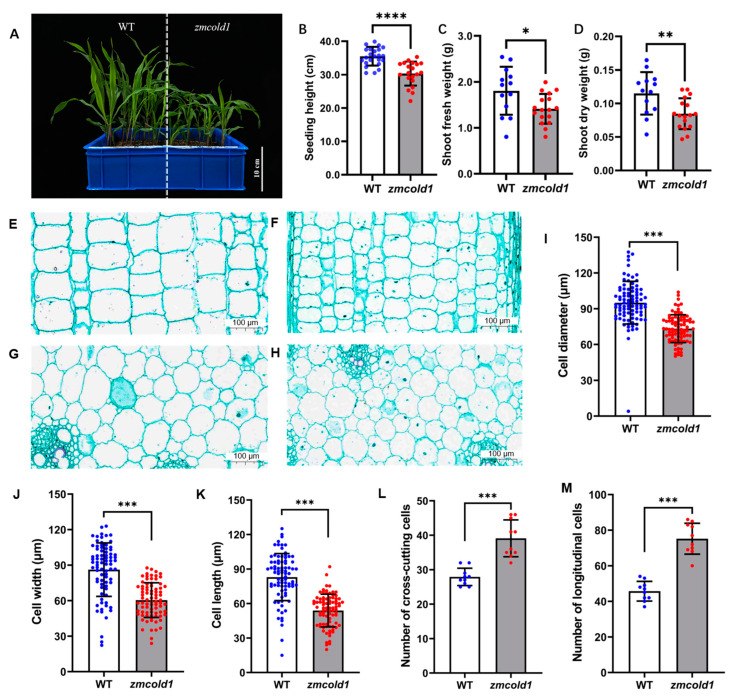
Dwarfism phenotype and cytological analysis of *zmcold1* mutant at the seedling stage. (**A**) Dwarfism phenotype at the seedling stage; scale bar, 10 cm. (**B**) Measurements of the plant height at seedling stage. (**C**) Fresh weight of above-ground part (**D**) Dry weight of above-ground part. (**E**) Longitudinal cutting of stem of KN5585 at the seedling stage; scale bar, 100 μm. (**F**) Stem lengthwise cut of *zmcold1* at the seedling stage; scale bar, 100 μm. (**G**) Transverse cutting of stem of KN5585; scale bar, 100 μm. (**H**) Transverse cutting of stem of *zmcold1*; scale bar, 100 μm. (**I**) Transverse cell diameter statistics. (**J**) Statistics on width of slitted cells. (**K**) Statistics on the length of slitted cells. (**L**) Statistics of the number of crosscut cells 0.126 mm^2^. (**M**) Statistics of 0.250 mm^2^ longitudinal cut cells. Asterisks indicate a significant difference compared to those of untreated controls by Student’s *t* test, * denote *p*-value < 0.05; ** denotes *p*-value < 0.01; *** denotes *p*-value < 0.001; **** denotes *p*-value < 0.0001.

To investigate the biological function of the *ZmCOLD1* gene, we generated one knock-out mutant by CRISPR-Cas9 (Figure S2A). Sanger sequencing showed that a C-base deletion was in the GPHR-N domain, and an A-base insertion was in the ABA-GPCR domain of ZmCOLD1 protein, and prematurely terminates translation (Figure S2B). The expression changes of *ZmCOLD1* gene in *zmcold1* mutants were further examined by RT-qPCR experiments, and it was found that the expression of this gene was significantly reduced in *zmcold1* knock-out mutant (Figure S2C). The *zmcold1* mutant apparently displayed dwarf phenotype compared with the wide-type inbred KN5585 at the four-leaf stage (Figure 4A,B). Additionally, we found that the fresh and dry weights of aboveground parts at the seedling stage of *zmcold1* mutant were significantly smaller than those of the wild-type (Figure 4C,D).

To investigate the cytological basis underlying the dwarfism, histological analysis of young stems of the *zmcold1* mutant and the KN5585 at the six-leaf stage was conducted (Figure 4E–H). In transverse sections, the average cell diameter of *zmcold1* mutant was 73.24 μm, which was significantly lower than that of the wild-type at 90.04 μm (Figure 4I). In longitudinal sections of stems, the average cell width of *zmcold1* mutant was 60.42 μm and the average length was 53.84 μm, both of which were significantly lower than those of KN5585 (Figure 4J,K). In addition, the number of mutant cells per unit area in both transverse and longitudinal sections was significantly lower than that of wild-type cells (Figure 4L,M). Thus, the loss-of-function of *ZmCOLD1* leads to a larger number of cells per unit area in the stalk, but a smaller cell size, which results in dwarfing of the plant (Figure 4L,M).

**Figure 5:**
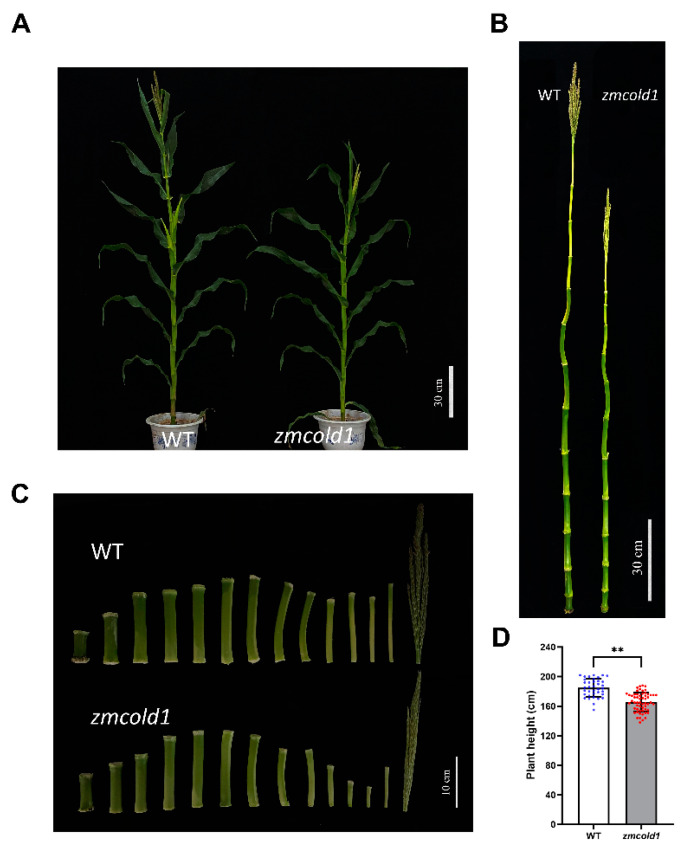
Plant height traits at grain maturity stage (June 2021, Hefei Anhui). (**A**,**B**) Wild-type and mutant plant height phenotype; scale bar, 30 cm. (**C**) Internode phenotype between wild-type and mutant; scale bar, 10 cm. (**D**) Plant height statistics. Data are mean ± s.d. (n = more than 35 biologically independent samples). Asterisks indicate a significant difference compared to those of untreated controls by Student’s *t* test, ** denotes *p*-value < 0.01.

**Figure 6:**
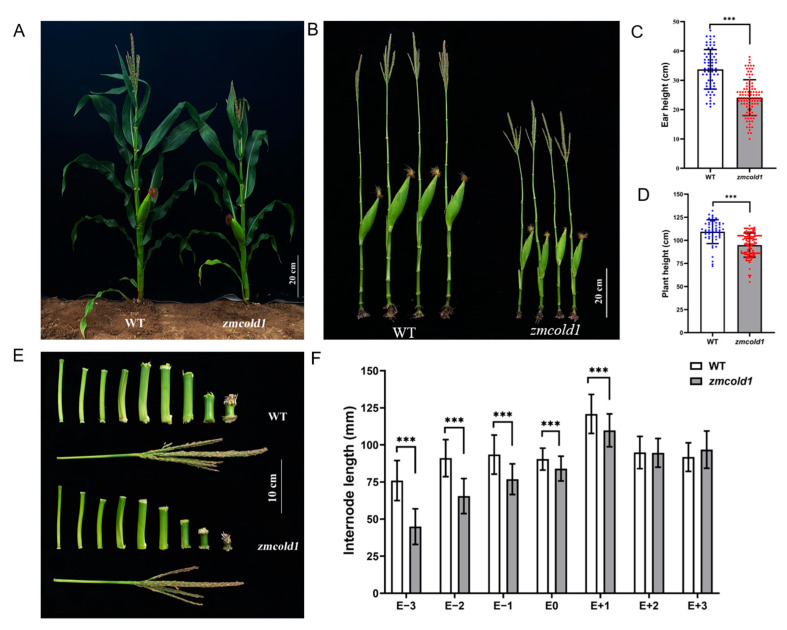
Plant height and ear height phenotype of maize at milk-ripe stage (Sanya, Hainan). (**A**,**B**) Wild-type and *zmcold1* plant phenotype; scale bars, 20 cm. (**C**) Panicle height statistics. Data are mean ± s.d. (n = more than 65 biologically independent samples). (**D**) Plant height statistics. Data are mean ± s.d. (n = more than 50 biologically independent samples). (**E**) Internode characters of mutant and wild-type; scale bar, 10 cm. (**F**) Statistics of internode length, E − 3~E + 3 respectively represent the third section under ear, the second section under ear, the first section under ear, the first section above ear, the second section above ear, and the third section above ear. Data are mean ± s.d. (n = more than 50 biologically independent samples). Asterisks indicate a significant difference compared to those of untreated controls by Student’s *t* test, *** denotes *p*-value < 0.001.

Subsequently, the phenotypes of the *zmcold1* mutant at maturity stage in the field were observed at two locations. The plant height of the *zmcold1* mutant showed the consistent dwarfing phenotype both in Hefei (Figure 5) and Sanya (Figure 6). The plant height of *zmcold1* mutant was significantly lower than those of KN5585 (Figure 5A,D). In addition, it can be observed that the internodes of the uppermost ear, as well as the internode below the uppermost ear of the mutant were apparently shorter than those of the wild-type (Figure 5B,C). In Sanya, more agronomical traits were measured. Firstly, the plant height, as well as the spike position of *zmcold1*, were significantly lower than those of KN5585 (Figure 6A–D). Additionally, the three internodes below and the first internode above the uppermost ear were significantly shorter in the mutant plants that those in KN5585 (Figure 6E,F). It is suggested that the mutation of *ZmCOLD1* caused the shortening of the three internodes length below and the first internode below the uppermost ear, as well as the internode of the uppermost ear, which in turn led to the dwarfing of the maize *zmcold1* mutant plants. Collectively, these results revealed that *ZmCOLD1* serves as an important regulator of plant height in maize.

**Figure 7:**
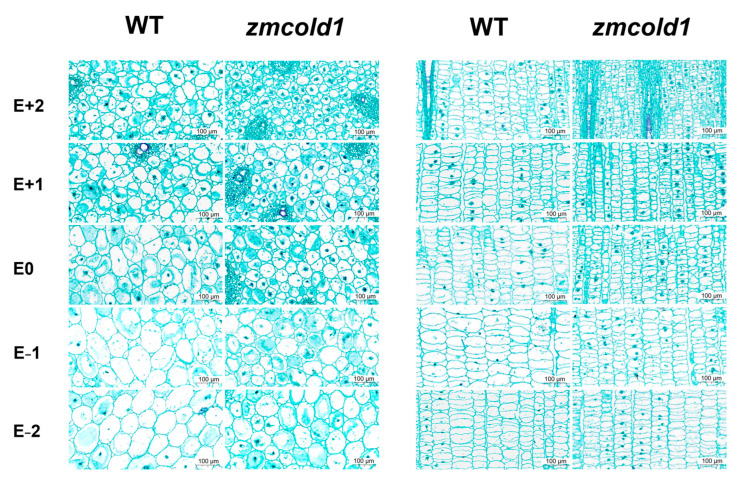
Cytological observation of internode sections at tasseling stage (January 2022, Sanya Hainan). E − 3~E + 3 respectively represent the third section under ear, the second section under ear, the first section under ear, the first section above ear, the second section above ear, and the third section above ear. Scale bars, 100 μm.

Finally, histological analysis of stems of the *zmcold1* mutant and the KN5585 at the tasseling stage was comprehensively conducted. The number of cells per unit area was significantly higher in both transverse sections and longitudinal sections of the *zmcold1* mutant than that in KN5585 (Table 1 and Figure 7). Further measurement of cell size revealed that the cell diameter of *zmcold1* mutants was reduced by 25% to 45% (transverse sections), and both cell length and width were reduced by 25% to 45% (longitudinal sections). Thus, cytological morphological observations further confirmed that the increased cell number but significantly smaller cell size in the *zmcold1* mutant resulted in dwarfism at the maturity stage.

### 2.4. ZmCold1 Regulates Seed Development

**Figure 8:**
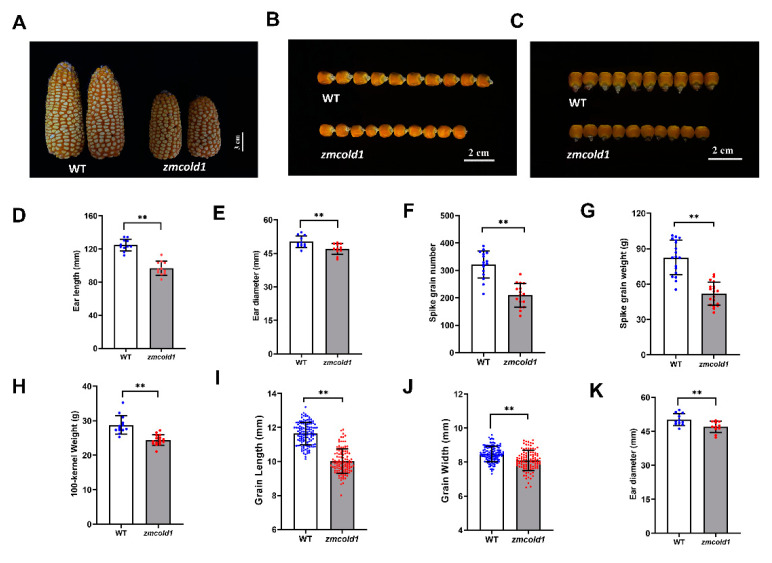
Phenotypes of ear and agronomics traits of grain for *zmcold1* (March 2022, Sanya Hainan). (**A**) Ear phenotype of WT and *zmcold1*; scale bar, 3 cm. (**B**,**C**) Seed of wild-type and *zmcold1*, (n = 10 biologically independent samples); scale bars, 2 cm. (**D**) Ear length of wild-type and *zmcold1*; data are mean ± s.d. (n = 11 biologically independent samples). (**E**) Ear width of wild-type and *zmcold1*; data are mean ± s.d. (n = 11 biologically independent samples). (**F**) Grain count per spike of wild-type and *zmcold1*; data are mean ± s.d. (n = 16 biologically independent samples). (**G**) Grain weight per spike of wild-type and *zmcold1*; data are mean ± s.d. (n = 19 biologically independent samples). (**H**) 100-kernel weight of WT and *zmcold1* seeds; data are mean ± s.d. (n = 13 and 16 biologically independent samples, respectively). (**I**) Grain length of wild-type and *zmcold1*; data are mean ± s.d. (n = 124 and 112 biologically independent samples, respectively). (**J**) Grain width of wild-type and *zmcold1*; data are mean ± s.d. (n = 125 and 112 biologically independent samples, respectively). (**K**) Grain thickness of wild-type and *zmcold1*; data are mean ± s.d. (n = 125 and 112 biologically independent samples, respectively). Asterisks indicate a significant difference compared to those of untreated controls by Student’s *t* test, ** denotes *p*-value < 0.01.

Yield related agronomic traits merit more attention. When the seeds matured, we found that the spikelet of *zmcold1* mutant were slightly shortened and thinner, and the width of the seeds was apparently reduced (Figure 8A–C and Figure S3A–C). Thus, in this study, the agronomic traits of *zmcold1* mutant seeds, including ear length, ear diameter, spike grain number, spike grain weight, 100-kernel weight, grain length, grain width, and ear diameter, was measured (Figure 8D–K and Figure S3D–G). The results showed that *zmcold1* mutant spikelet were full, and spikelet length, diameter, number of grains, and spikelet weight were significantly smaller than those of the KN5585. The average grain length and width of *zmcold1* mutant were 10.02 mm and 8.096 mm, respectively, which were significantly lower than those of the wild-type. More importantly, the 100-seed weight of zmcold1 mutant was 24.40 g, which was significantly lower than that of the wild-type, indicating that *ZmCOLD1* plays an important role in regulation of seed development (Figure 8 and Figure S3).

### 2.5. Knock-Out of ZmCOLD1 Enhances ABA Sensitivity of Maize

**Figure 9:**
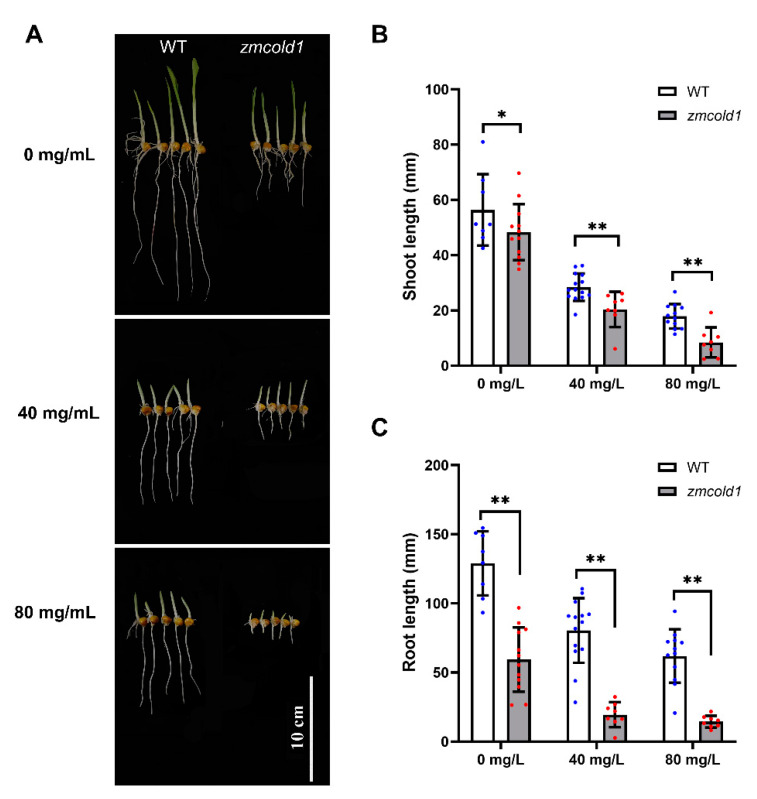
*zmcold1* mutant exhibited remarkable sensitivity to ABA after germination. (**A**) Effects of different concentrations of ABA on seed germination and growth; scale bar, 10 cm. (**B**) Statistics of different concentrations of ABA on bud growth; data are mean ± s.d. (n = more than 8 biologically independent samples). (**C**) Statistics on root growth of different ABA concentrations; data are mean ± s.d. (n = more than 8 biologically independent samples). Asterisks indicate a significant difference compared to those of untreated controls by Student’s *t* test, * denotes *p*-value < 0.05; ** denotes *p*-value < 0.01.

To decipher whether *ZmCOLD1* plays a role in the ABA signaling pathway in maize, ABA sensitivity analysis was conducted by seed germination experiment. Seeds of wild-type and *zmcold1* mutant plant were germinated, treated with 0, 40, and 80 mg/L ABA solution, respectively. The shoot length and root length of the seeds were observed and estimated after 5 days. In the absence of ABA treatment (0 mg/L ABA), the shoot length of *zmcold1* was not significantly different from that of the wild-type, but the root length of the *zmcold1* mutant was significantly shorter by 53.82%, indicating that root elongation was affected by the mutation of *ZmCOLD1* gene. In contrast, the root of *zmcold1* mutant plants showed significant ABA sensitivity after treatment with different concentrations of ABA at 40 mg/L and 80 mg/L, and their root elongation was significantly inhibited by ABA (Figure 9).

### 2.6. ZmCOLD1 Interact with ZmCT2 via Hydrophilic Loop

**Figure 10:**
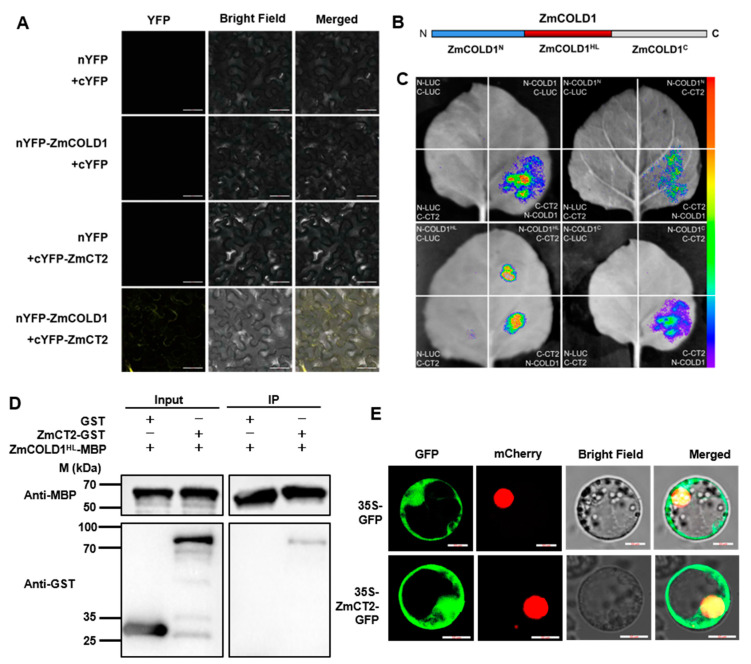
The hydrophilic loop of ZmCOLD1 physically associate with ZmCT2. (**A**) BiFC assay was conducted in leaves of *N. benthamiana* to test the protein interaction between ZmCOLD1 and ZmCT2. The YFP fluorescence signals were detected 48 h post infiltration (hpi). BF, bright field. Scale bars, 20 μm. (**B**) Diagrams of the ZmCOLD1 derivatives. (**C**) LCA assays illustrated that ZmCOLD1^HL^ associates with ZmCT2 in *N. benthamiana* leaves. Empty vectors were used as negative controls. (**D**) Pull-down assay showed the interaction between ZmCOLD1^HL^ and ZmCT2. Anti-MBP and anti-GST antibodies were used for the immunoblotting. (**E**) Subcellular co-localization of ZmCOLD1 with PM131 marker in maize mesophyll protoplasts. GFP signal of ZmCOLD1-GFP was merged with RFP of NLS marker in the NLS. Scale bars, 10 μm.

The orthologous genes of ZmCOLD1 in *Arabidopsis*, rice and wheat, was reported to interact with the Gα protein. In the present study, the canonical Gα protein (RGA1), ZmCT2, was identified and cloned from maize. ZmCT2 harbored a G-alpha conserved domain, a GTP/Mg^2+^ binding site as well as a potential receptor binding site. To further verify whether the ZmCOLD1 protein physically interacts with ZmCT2, we performed bimolecular fluorescence complementation (BiFC) and firefly luciferase complementation imaging (LCI) assays (Figure 10A,C). BiFC assays showed reconstituted YFP fluorescence could be detected in the plasma membrane of tobacco transiently co-expressing nYFP-ZmCOLD1 and cYFP-ZmCT2. However, no YFP fluorescence could be detected in the negative controls (NYFP-ZmCOLD1 and CYFP, NYFP and CYFP-ZmCT2) (Figure 10A). In LCI assay, ZmCOLD1 was fused with the nLUC and ZmCT2 was fused with cLUC. Results showed that strong luminescence signals could be identified in nLUC-ZmCOLD1 and cLUC-ZmCT2 co-expression tobacco leaves, whereas no signal could be detected in those negative controls (Figure 10C). These results demonstrated that ZmCOLD1 could interact with ZmCT2 in vivo.

Additionally, it has been shown that the central hydrophilic loop (HL, residues 178–307) domain in the TaCOLD1 protein is a key site for the interaction with TaGα-7A protein [17]. To verify whether the same functional domain exists in ZmCOLD1, we segmented ZmCOLD1 into three segments: ZmCOLD1^N^, ZmCOLD1^HL^, and ZmCOLD1^C^ (Figure 10B). LCI assays were applied to prove that the HL domain fragment of ZmCOLD1, ZmCOLD1^HL^, was required for interaction with ZmCT2, while the C-terminus and N-terminus of ZmCOLD1 were not (Figure 10C). In addition, pull-down assay repeatedly exhibited GST-ZmCT2 could be pulled down by MBP-ZmCOLD1^HL^, demonstrating that ZmCOLD1^HL^ protein binds to ZmCT2 protein in vitro (Figure 10D). Furthermore, ZmCT2 protein fused with GFP was transiently expressed in maize protoplasts. The results showed that the fusion protein of ZmCT2-GFP was expressed on the plasma membrane, which was in line with the subcellular location of ZmCOLD1 (Figure 10E). These results demonstrated that ZmCOLD1 protein interacts with the ZmGα protein ZmCT2 and the HL domain of ZmCOLD1 is dispensable for the interaction between ZmCOLD1 and ZmCT2.

### 2.7. Transcriptome Analysis of zmcold1 Mutant

**Figure 11:**
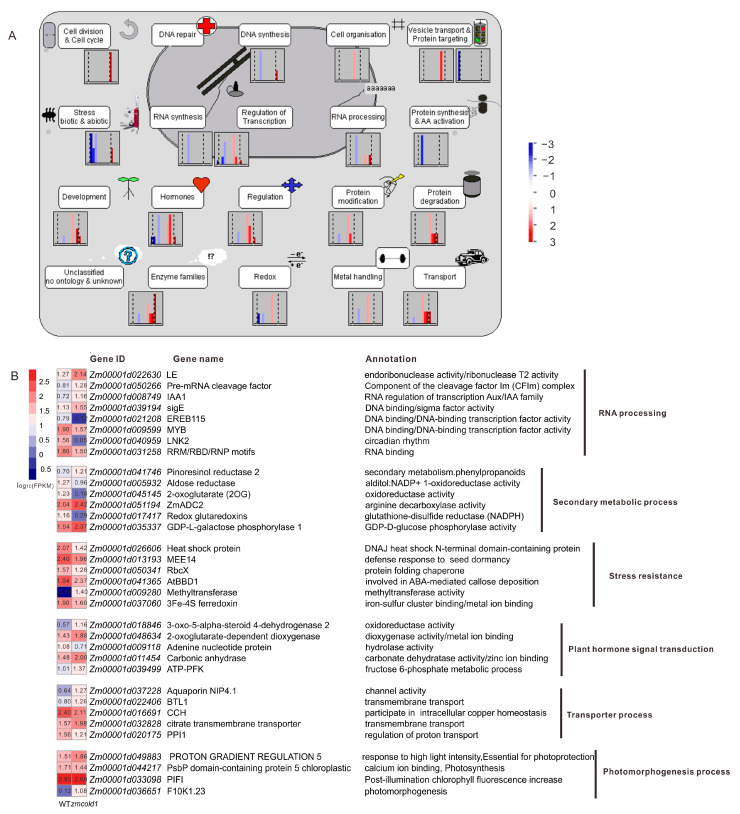
Functional Annotation Analysis of DEGs. (**A**) Functional Annotation Analysis of Mapman Pathway. (**B**) Heat map and annotations of representative genes related to RNA processing, secondary metabolic process, stress resistance, plant hormone signal transduction, transporter process, photomorphogenesis process. FPKM represents Fragments Per Kilobase of exon model per Million mapped fragments.

To further understand the mechanism of G protein-coupled receptor ZmCOLD1 in regulating plant height and seed development, high-throughput transcriptome was performed between the *zmcold1* mutant and wild-type plant in the present study. Consequently, a total of 212 differentially expressed genes (DEGs) were identified, among which are 143 up-regulated and 69 down-regulated DEGs (Figure S4A,B). Most of the DEGs are significantly enriched in the cellular pathways of growth and development, resistance, transcriptional regulation, hormones, and enzyme-related cellular pathways (Figure 11A). Functional annotation analysis DEGs showed that these genes were associated with RNA processing, secondary metabolism, adversity, phytohormone signaling, transport, and photomorphogenesis (Figure 11B). Among them, *DERB1A* is associated with maize cottonseed sugar synthase to control cottonseed sugar accumulation and plant cold resistance; *ADC2* encodes arginine decarboxylase associated with putrescine synthesis to improve plant cold tolerance; BIF1/AUX22 encodes serine/threonine protein kinase to regulate growth hormone transport and promote inflorescence cell growth; 3-oxo-5-alpha-steroid 4-dehydrogenase 2 is associated with oleuropein lactone, one of the important hormones involving in plant height; *KIN1* and bifunctional nuclease 1 are associated with plant ABA signaling pathway, which enhances plant stress tolerance as well as promotes stomatal closure; and *ZmNIP2* is related to nitrate transport. Thus, the loss-of-function of *ZmCOLD1* resulted in differential expression of many genes related to plant hormones and abiotic stresses, which indicated that *ZmCOLD1* may regulate plant development via coordinating the expression genes in multiple biological processes.

## 3. Discussion

Heterotrimeric G proteins are important signaling components found in eukaryotes, which regulate a series of physiological and biochemical responses in the cell by transmitting signals from transmembrane receptors to the cell [20]. Although a large number of studies have been conducted to show the function of heterotrimeric G proteins components in plants, there are seldom studies related to the GPCR-like proteins in maize. In the present study, the orthologous gene of *AtGTG1*/*GTG2* and *OsCOLD1* was identified from maize and named as *ZmCOLD1* (Figure 1). ZmCOLD1 harbored a RAS-GTPase active region and an ATP-/GTP binding region, similar to the case of OsCOLD1. ZmCOLD1 located in the ER and the plasma membrane (Figure 3), which is the same as its orthologous proteins, OsCOLD1 and TaCOLD1 [10,17]. Furthermore, ZmCOLD1 physically interacted with the heterotrimeric G protein Gα subunit, ZmCT2. Thus, we speculate that ZmCOLD1 is a GPCR-like protein and could combine extracellular signals with a Gα subunit.

Heterotrimeric G proteins system have crucial roles in multitude signaling and development pathways in plants, strongly suggesting the function diversity of the components of heterotrimeric G proteins [21,22,23,24]. OsCOLD1 protein was identified as a cold sensor and related to cold tolerance by activation of Ca^2+^ channels for sensing low temperature [10]. However, *TaCOLD1* in wheat is related to plant height and photoperiod [17]. The homologs of COLD1 in *Arabidopsis*, GTG1 and GTG2, are involved in light signaling [16]. Overexpression of *ZmCOLD1* gene in maize B104 elite line promotes chilling tolerance during seed germination [25]. In the present study, we found that ZmCOLD1 could affect plant height and seed development (Figure 5 and Figure 6). Additionally, the yield-related traits of the *zmcold1* mutant were significantly reduced, indicating the potential roles of *ZmCODL1* in seed development. ZmCOLD1 was also proved to physically interact with the maize Gα subunit, ZmCT2, through the HL domain. Previous reports demonstrated that ZmCT2 is part of a signaling pathway that controls the size of maize meristematic tissue, and that null mutations in ZmCT2 reduce above-ground growth, resulting in a dwarfed maize phenotype that exhibits coarser male ear branches [26,27,28]. In our results, cytological observation showed that the cell size in the stalks of maize in the *zmcold1* mutant has been reduced in both the seedling and adult stages, which is probably due to the fact that after the mutation of the ZmCOLD1 gene, ZmCT2, which interacts with ZmCOLD1, functionally regulates the plasticity in plant development.

The plant hormone ABA mediates diverse physiological and developmental processes [29]. The mechanism underlying ABA signal transduction is a fundamental question [30,31]. Heterotrimeric G protein complex is important in ABA signaling in plants [32,33,34,35]. In *Arabidopsis*, the Gα subunit GPA1 regulates stomatal response and seed germination in an ABA-dependent manner [32]. GPCR proteins, GCR1 and GCR2, could interact with GPA1 and regulate ABA signaling transduction [8,29]. GCR2 could bind ABA and was demonstrated to be a plasma membrane receptor for ABA. The seeds of GCR2-overexpressing plants were hypersensitive to ABA [33]. The homologs of COLD1 in *Arabidopsis*, GTG1 and GTG2, are involved in ABA signaling by participating in G protein-coupled ABA signaling and are part of the ABA receptor complex, thus participating in ABA signaling [2]. In this study, the *zmcold1* mutant was hypersensitive to ABA in seed germination experiments and could interact with ZmCT2 (Figure 9 and Figure 10).

The current research provides new evidence that *ZmCOLD1* plays an important role in regulation plant height and seed development. However, the molecular mechanism and genetic relationship between ZmCOLD1 and ZmCT2 need to be clarified in the future. Furthermore, genes involved in multiple pathways were differentially expressed between *zmcold1* mutant plant and KN5585. More effort will be made to illustrate the key pathways that act downstream of ZmCOLD1, particularly those genes that were proposed to be involved in the regulation of cell size. According to a precious highlighted research, overexpression of *ZmCOLD1* leads to enhanced germination rate under chilling stress, which shows similar tendency as OsCOLD1. However, loss-of-function of *ZmCOLD1* in maize inbred line KN5585 caused dwarfism and small kernels because of reduced cell size, which demonstrated that ZmCOLD1 is dispensable in regulation of growth and seed development. The present study modestly extends our knowledge about GPCR-like protein in maize.

## 4. Materials and Methods

### 4.1. The Plant Materials and Growth Conditions

To create the knock-out mutant plants of *ZmCOLD1*, the guide RNA was designed to target the exon of *ZmCOLD1*, which were cloned into PCXB053 vector under the drive of ZmU6-1 promoter. The constructs were then introduced into *Agrobacterium* strain EHA105 and transformed into immature embryos of inbred line KN5585 through an *Agrobacterium*-mediated transformation system. The *zmcold1* mutant transgenic line were generated at Weimi Bio-tech Company (Changzhou, Jiangsu, China). The obtained mutant plants were crossed with KN5585 and further self-pollinated to generate homozygous plant. Afterwards, the lines without Cas9 were further screened by PCR amplification and Sanger sequencing. Both of the knock-out mutant plants of *ZmCOLD1* and KN5585 plants were cultivated to evaluate the agronomical traits in April of 2021 (Hefei city) and in November of 2021 (Sanya city) under normal field management.

### 4.2. RNA Extraction and Quantitative RT-PCR Analysis

To evaluate the expression profiles of *ZmCOLD1*, samples of B73 inbred line were collected and immediately frozen in liquid nitrogen. The total RNA was isolated with TRIzol reagent (AG21101, Accurate Biology, Changsha, Hunan, China). cDNA was synthesized with 2 µg of total RNA using the 5×All-In One RT MasterMix system (AG21101, Accurate Biology, Changsha, Hunan, China) according to the manufacturer’s instructions. Afterwards, the cDNA was diluted with five-fold distilled water. FastStart™ Universal SYBR^®^ Green Master (Roche, Basel, Switzerland) was then used for quantitative RT-PCR assays according to a previous report [36]. Each experiment was repeated with three biological replicates. The raw dataset was further dealt with the 2_DDCt method. Additionally, the expression level of *ZmACTIN* was used as internal control to normalize the expression levels of target genes. All the primers used for RT-qPCR assays are listed in Table S1.

### 4.3. Generation of DNA Constructs

Both pCAMBIA1300-nLuc and pCAMBIA1300-cLuc vectors were used in a luciferase complementation assay (LCA). Coding sequences of *ZmCOLD1*, *ZmCOLD1^HL^* or *ZmCT2* were inserted into pCAMBIA1300-nLUC and pCAMBIA1300-cLUC, respectively, to generate ZmCOLD1-nLUC, ZmCOLD1^HL^-nLUC, and ZmCT2-cLUC vectors. In brief, the amplified target genes were separately cloned into the *Kpn* I digested pCAMBIA1300-nLUC and *Sal* I digested pCAMBIA1300-cLUC vectors [37]. The construct, expressing NLS-mCherry, was used as a control for protoplast co-transfection and nucleus labelling. PM131 is cell membrane localization marker; ER is a localization marker for endoplasmic reticulum. To construct vectors for subcellular localization, the coding sequence of *ZmCOLD1* and *ZmCT2* were PCR amplified using gene-specific primers and inserted into d35S-GFP-NOS plasmid. For pull-down assays, the constructs were based on the vectors pMAL-c4X and pGEX-4T-1. Briefly, the central hydrophilic loop of *ZmCOLD1* (residues 178–307) was cloned into *Eco*R I digested pMAL-c2X to generate MBP-ZmCOLD1^HL^; the coding sequence of *ZmCT2* was cloned into *Bam*H I-digested pGEX-4T-1 to generate GST-ZmCT2. To generate ZmCOLD1-YN and ZmCT2-YC constructs in a biomolecular florescence complementation (BiFC) assay, coding sequence of *ZmCOLD1* or *ZmCT2* were cloned into pUC-SPYNE and pUC-SPYCE vectors [38], respectively. The sequences of these primers are listed in Supplemental Table S1.

### 4.4. RNA In Situ Hybridization Assays

The shoot apical meristem tissues of B73 inbred line at V4 stage was collected to conduct RNA in situ hybridization. The anti-sense probe of *ZmCOLD1* was designed (CTCCAATCACCCCAATCCTACTGACCAACTGC) and labeled by SweAMI kit (GDP1087, Servicebio, Wuhan, Hubei, China.). RNA in situ hybridization was performed according to the previous report [39].

### 4.5. Subcellular Localization Analysis in Maize Protoplasts

The full-length coding sequence of *ZmCOLD1* and *ZmCT2* were cloned and fused in the frame with green fluorescent proteins (GFP) in the binary vector pCAMBIA1305-GFP, respectively. The construct, expressing NLS-mCherry, was used as a control for protoplast co-transfection and nucleus labelling. The recombinant vectors were transformed into maize mesophyll protoplasts, according to methods as described previously [40]. After 36 h of incubation under dark at 24 °C, protoplasts were examined by a confocal microscopy (Car Zeiss, LSM880, Jena, Germany).

### 4.6. Protein Expression and In Vitro Pull-Down Assay

To test the protein–protein interaction between ZmCOLD1^HL^ and ZmCT2, the central hydrophilic loop of *ZmCOLD1* was cloned into the pMal-c4X vector, using *Eco*R I restriction sites, to generate a fusion construct of ZmCOLD1^HL^ with Maltose Binding Protein (MBP-ZmCOLD1^HL^). Coding sequence of ZmCT2 was cloned into pGEX-4T-1 to produce GST-ZmCT2 plasmid. Recombinant proteins were induced by 0.5 mM β-D-1-thiogalactopyranoside (IPTG) for 16 h in *E. coli* Rosetta DE3 competent cells (No. CMC0014, Sigma-Aldrich, Shanghai, China). The proteins were purified using Glutathione Sepharose beads (Sangon Biotech, Shanghai, China) or Amylose Resin (No. E8021L, New England Biolabs Inc., Ipswich, MA, USA). Approximately, 1 μg MBP protein, as well as MBP- ZmCOLD1^HL^ recombinant protein were incubated in 50 μL MBP beads in MBP-binding buffer at 4 °C for 2 h, respectively. Afterwards, 1 μg GST-ZmCT2 recombinant protein were added and incubated overnight at 4 °C. Subsequently, the mixture was washed three times with TGH buffer (150 mM NaCl, 1.5 mM MgCl_2_, 1 mM EGTA, 1% triton X-100, 10% glycerol, protease inhibitor, and 50 mM HEPES; pH 7.5) for 10 min. Cleared proteins were further eluted by boiling in 1 × SDS loading buffer, separated using SDS-PAGE. Protein was transferred to PVDF (Amersham Hybond, 0.45 μm) in transfer buffer (0.025 M Tris, 0.192 M glycine, 20% alcohol (*v*/*v*) by BIORAD 165-8001 for 12 h. Eventually, proteins were incubated with anti-GST or anti-MBP in 100 μL antibody pretreat solution (HRP/Mouse) and detected by ChemiDocTM MP Imaging System (BIO-RAD, Singapore).

### 4.7. Luciferase Complementation Assay

Luciferase complementation assay was conducted as described previously [37]. The full-length CDS and truncated version of *ZmCOLD1* sequence were fused with N-terminal half of luciferase to generate ZmCOLD-nLUC and ZmCOLD1^HL^-nLUC, respectively. The full-length CDS of *ZmCT2* was inserted into C-terminal half of luciferase cLUC. Constructed plasmid was transferred into Agrobacterium GV3101 cells. Different combinations were then co-transfected into the *N. benthamiana* leaves. After being kept in darkness for 48 h, LUC signals were detected with leaves sprayed with 0.5 mM luciferin and imaged using in vivo plant imaging system (NightShade LB985, Berthold, Germany). 

### 4.8. Bimolecular Fluorescence Complementation (BiFC) Assay

To confirm the interaction between ZmCOLD1 and ZmCT2, full-length CDS of *ZmCOLD1* sequence was cloned into pUC-SPYCE vector harboring the N-terminus of eYFP to generate nYPF-ZmCOLD1 construct [41,42]. The full-length CDS of *ZmCT2* was ligated to the pUC-SPYNE vector containing the C-terminus of eYFP to construct cYFP-ZmCT2 plasmids. Equal amounts of the mixed plasmids were transformed into the maize mesophyll protoplasts. The YFP signals were detected using an excitation wavelength of 488 nm on a confocal microscope (Zeiss LSM800, Jena, Germany).

### 4.9. RNA-Seq and Data Analysis Pipeline

The maize inbred line KN5585 and *zmcold1* mutant plants were grown in growth chambers (PERCIVAL AR-41L3, Iowa, America) with light intensity of 300 µmol m^2^ s^−1^ field under a 16:8 (L:D) photoperiod. Leaves of three seedlings with considerable growth vigor were separately collected as three biological replicates, which were then frozen immediately in liquid nitrogen. Total RNA was extracted using TRIzol reagent (Invitrogen, Carlsbad, CA, USA), then subjected to high throughput sequencing by Grand Omics (Wuhan, Hubei, China). Trimmomatic software (Version 0.39) was employed to filter low-quality and adaptor sequences [43]. Hisat2 software (Version 2.0) [44] was utilized to align clean data to the maize B73 reference genome [45]. The resulting BAM files were piped into StringTie (version 2.1.5) [46] and fragments per kilobase of transcript per million mapped reads (FPKM) values were calculated to evaluate the expression level. DESeq packages (version 2.0) [47] was used to determine differentially expressed genes (DEGs) and the *p*-values was adjusted by Benjamini–Hochberg false discovery correction. The following criterions was applied to identify DEGs between KN5585 and *zmcold1* mutant plants: (1) The fold-change of gene expression was larger than 2.0 or smaller than 0.5; (2) the adjusted *p*-value was less than 0.05; (3) FPKM value in at least one biological replicate was larger than 1.0. The Mapman software (Version 3.7.0) was further used to map the expression profiles of DEGs onto diverse metabolic pathways [48].

## 5. Conclusions

The present research aimed to identify the GPCR-like protein in maize and decipher its biological function, as well as the molecular mechanism. As a result, ZmCOLD1, a GPCR-like protein, was identified from maize by blast research. ZmCOLD1 was orthologous with OsCOLD1, AtGTG1, and AtGTG2. ZsmCOLD1 protein was proved to be localized to the cell membrane and endoplasmic reticulum by transient expression. Our results showed that knock-out of *ZmCOLD1* results in dwarf phenotype. Histological analysis highlights that *ZmCOLD1* gene regulate plant height development by affecting the cell division process, and the increased cell number but significantly smaller cell size may result to dwarfing of *zmcold1*. Another major finding is that ZmCOLD1 could interact with the maize Gα subunit, ZmCT2, though the central hydrophilic loop domain, in vivo and in vitro. ABA sensitivity analysis by seed germination assays exhibited that *zmcold1* were hypersensitive to ABA, indicating its important roles in ABA signaling. The present study provides a framework for the functional investigation of GPCR-like proteins in maize.

## Figures and Tables

**Table 1:** Statistics of characteristics of cells in the internode sections.

Position	Traits	WT	*zmcold1*	The Difference Between Zmcold1 and WT (±%)
**E + 2**	Number of cells of cross section	37 ± 3	66 ± 4	+78.38% **
	Number of cells of longitudinal section	69 ± 7	94 ± 5	+36.23% **
	Diameter of cells of cross section (μm)	67.235 ± 8.076	50.595 ± 6.642	−24.75% **
	Length of cells of longitudinal section (μm)	29.273 ± 6.444	21.89 ± 4.749	−25.22% **
	Width of cells of longitudinal section (μm)	59.19 ± 7.312	41.981 ± 4.770	−29.07% **
**E + 1**	Number of cells of cross section	33 ± 3	41 ± 4	+24.24% **
	Number of cells of longitudinal section	58 ± 3	82 ± 6	+41.38% **
	Diameter of cells of cross section (μm)	79.955 ± 11.418	64.168 ± 8.650	−19.74% **
	Length of cells of longitudinal section (μm)	31.705 ± 6.698	18.218 ± 5.028	−42.54% **
	Width of cells of longitudinal section (μm)	80.482 ± 10.429	43.862 ± 5.228	−45.50% **
**E0**	Number of cells of cross section	24 ± 3	46 ± 6	+91.67% **
	Number of cells of longitudinal section	46 ± 6	88 ± 8	+91.30% **
	Diameter of cells of cross section (μm)	103.286 ± 12.730	69.586 ± 7.677	−32.63% **
	Length of cells of longitudinal section (μm)	40.327 ± 8.535	23.595 ± 6.137	−41.49% **
	Width of cells of longitudinal section (μm)	93.086 ± 13.041	47.641 ± 8.138	−48.82% **
**E − 1**	Number of cells of cross section	17 ± 1	30 ± 3	+76.47% **
	Number of cells of longitudinal section	37 ± 5	58 ± 6	+56.76% **
	Diameter of cells of cross section (μm)	133.923 ± 13.901	82.614 ± 8.042	−38.31% **
	Length of cells of longitudinal section (μm)	51.473 ± 11.077	28.195 ± 6.664	−45.22% **
	Width of cells of longitudinal section (μm)	119.073 ± 10.944	66.955 ± 10.544	−43.77% **
**E − 2**	Number of cells of cross section	22 ± 2	27 ± 2	+22.73% **
	Number of cells of longitudinal section	55 ± 4	64 ± 7	+16.36% **
	Diameter of cells of cross section (μm)	111.809 ± 12.203	84.75 ± 10.474	−24.20% **
	Length of cells of longitudinal section (μm)	30.990 ± 5.281	20.905 ± 6.111	−32.54% **
	Width of cells of longitudinal section (μm)	85.395 ± 15.855	63.4 ± 10.738	−25.76% **

E0 represents the internode of the uppermost ear; Note: ±SE; ** represents *p*< 0.01 (student-*t* test).

## Data Availability

The raw sequencing dataset from this article has been submitted to the National Genomics Data Center at the Beijing Institute of Genomics, under accession number CRA019838.

## Supplementary Materials

The following supporting information can be downloaded at: https://www.mdpi.com/article/10.3390/ijms252111755/s1

## References

[1] Mohanasundaram B., Pandey S. (2023). Moving beyond the Arabidopsis-Centric View of G-Protein Signaling in Plants. Trends Plant Sci..

[2] Pandey S., Nelson D.C., Assmann S.M. (2009). Two Novel GPCR-Type G Proteins Are Abscisic Acid Receptors in Arabidopsis. Cell.

[3] Ofoe R. (2021). Signal Transduction by Plant Heterotrimeric G-Protein. Plant Biol. Stuttg. Ger..

[4] Chen J.-G. (2008). Heterotrimeric G-Proteins in Plant Development. Front. Biosci. J. Virtual Libr..

[5] Zhang H., Xie P., Xu X., Xie Q., Yu F. (2021). Heterotrimeric G Protein Signalling in Plant Biotic and Abiotic Stress Response. Plant Biol. Stuttg. Ger..

[6] Oldham W.M., Hamm H.E. (2008). Heterotrimeric G Protein Activation by G-Protein-Coupled Receptors. Nat. Rev. Mol. Cell Biol..

[7] McCudden C.R., Hains M.D., Kimple R.J., Siderovski D.P., Willard F.S. (2005). G-Protein Signaling: Back to the Future. Cell. Mol. Life Sci..

[8] Pandey S., Assmann S.M. (2004). The Arabidopsis Putative G Protein-Coupled Receptor GCR1 Interacts with the G Protein Alpha Subunit GPA1 and Regulates Abscisic Acid Signaling. Plant Cell.

[9] Chakraborty N., Raghuram N. (2023). Life, Death and Resurrection of Plant GPCRs. Plant Mol. Biol..

[10] Ma Y., Dai X., Xu Y., Luo W., Zheng X., Zeng D., Pan Y., Lin X., Liu H., Zhang D. (2015). COLD1 Confers Chilling Tolerance in Rice. Cell.

[11] Torres-Rodriguez M.D., Lee S.G., Roy Choudhury S., Paul R., Selvam B., Shukla D., Jez J.M., Pandey S. (2024). Structure-Function Analysis of Plant G-Protein Regulatory Mechanisms Identifies Key Gα-RGS Protein Interactions. J. Biol. Chem..

[12] Johnston C.A., Taylor J.P., Gao Y., Kimple A.J., Grigston J.C., Chen J.-G., Siderovski D.P., Jones A.M., Willard F.S. (2007). GTPase Acceleration as the Rate-Limiting Step in Arabidopsis G Protein-Coupled Sugar Signaling. Proc. Natl. Acad. Sci. USA.

[13] Urano D., Jones J.C., Wang H., Matthews M., Bradford W., Bennetzen J.L., Jones A.M. (2012). G Protein Activation without a GEF in the Plant Kingdom. PLoS Genet..

[14] Urano D., Phan N., Jones J.C., Yang J., Huang J., Grigston J., Taylor J.P., Jones A.M. (2012). Endocytosis of the Seven-Transmembrane RGS1 Protein Activates G-Protein-Coupled Signalling in Arabidopsis. Nat. Cell Biol..

[15] Jones J.C., Duffy J.W., Machius M., Temple B.R.S., Dohlman H.G., Jones A.M. (2011). The Crystal Structure of a Self-Activating G Protein Alpha Subunit Reveals Its Distinct Mechanism of Signal Initiation. Sci. Signal..

[16] Jaffé F.W., Freschet G.-E.C., Valdes B.M., Runions J., Terry M.J., Williams L.E. (2012). G Protein-Coupled Receptor-Type G Proteins Are Required for Light-Dependent Seedling Growth and Fertility in Arabidopsis. Plant Cell.

[17] Dong H., Yan S., Liu J., Liu P., Sun J. (2019). TaCOLD1 Defines a New Regulator of Plant Height in Bread Wheat. Plant Biotechnol. J..

[18] Wang W., Guo W., Le L., Yu J., Wu Y., Li D., Wang Y., Wang H., Lu X., Qiao H. (2023). Integration of High-Throughput Phenotyping, GWAS, and Predictive Models Reveals the Genetic Architecture of Plant Height in Maize. Mol. Plant.

[19] Jin Y.-N., Cui Z., Ma K., Yao J.-L., Ruan Y.-Y., Guo Z.-F. (2021). Characterization of ZmCOLD1, Novel GPCR-Type G Protein Genes Involved in Cold Stress from Zea Mays L. and the Evolution Analysis with Those from Other Species. Physiol. Mol. Biol. Plants.

[20] Zou W., Yu Q., Ma Y., Sun G., Feng X., Ge L. (2024). Pivotal Role of Heterotrimeric G Protein in the Crosstalk between Sugar Signaling and Abiotic Stress Response in Plants. Plant Physiol. Biochem. PPB.

[21] Ferrero-Serrano Á., Chakravorty D., Kirven K.J., Assmann S.M. (2024). Oryza CLIMtools: A Genome–Environment Association Resource Reveals Adaptive Roles for Heterotrimeric G Proteins in the Regulation of Rice Agronomic Traits. Plant Commun..

[22] Ashikari M., Wu J., Yano M., Sasaki T., Yoshimura A. (1999). Rice Gibberellin-Insensitive Dwarf Mutant Gene Dwarf 1 Encodes the Alpha-Subunit of GTP-Binding Protein. Proc. Natl. Acad. Sci. USA.

[23] Pandey S. (2024). Agronomic Potential of Plant-Specific Gγ Proteins. Physiol. Mol. Biol. Plants Int. J. Funct. Plant Biol..

[24] Chaya G., Segami S., Fujita M., Morinaka Y., Iwasaki Y., Miura K. (2022). OsGGC2, Gγ Subunit of Heterotrimeric G Protein, Regulates Plant Height by Functionally Overlapping with DEP1 in Rice. Plants.

[25] Zhou Y., Zhang H., Zhang S., Zhang J., Di H., Zhang L., Dong L., Lu Q., Zeng X., Liu X. (2023). The G Protein-Coupled Receptor *COLD1* Promotes Chilling Tolerance in Maize during Germination. Int. J. Biol. Macromol..

[26] Wu Q., Regan M., Furukawa H., Jackson D. (2018). Role of Heterotrimeric Gα Proteins in Maize Development and Enhancement of Agronomic Traits. PLoS Genet..

[27] Urano D., Jackson D., Jones A.M. (2015). A G Protein Alpha Null Mutation Confers Prolificacy Potential in Maize. J. Exp. Bot..

[28] Bommert P., Je B.I., Goldshmidt A., Jackson D. (2013). The Maize Gα Gene COMPACT PLANT2 Functions in CLAVATA Signalling to Control Shoot Meristem Size. Nature.

[29] Hernández P.M., Arango C.A., Kim S.K., Jaramillo-Botero A., Goddard W.A. (2023). Predicted Three-Dimensional Structure of the GCR1 Putative GPCR in Arabidopsis Thaliana and Its Binding to Abscisic Acid and Gibberellin A1. J. Agric. Food Chem..

[30] Xie S., Luo H., Huang W., Jin W., Dong Z. (2024). Striking a Growth–Defense Balance: Stress Regulators That Function in Maize Development. J. Integr. Plant Biol..

[31] Guo J., Yang X., Weston D.J., Chen J.-G. (2011). Abscisic Acid Receptors: Past, Present and Future. J. Integr. Plant Biol..

[32] Wang X.Q., Ullah H., Jones A.M., Assmann S.M. (2001). G Protein Regulation of Ion Channels and Abscisic Acid Signaling in Arabidopsis Guard Cells. Science.

[33] Liu X., Yue Y., Li B., Nie Y., Li W., Wu W.-H., Ma L. (2007). A G Protein-Coupled Receptor Is a Plasma Membrane Receptor for the Plant Hormone Abscisic Acid. Science.

[34] Subramaniam G., Trusov Y., Lopez-Encina C., Hayashi S., Batley J., Botella J.R. (2016). Type B Heterotrimeric G Protein γ-Subunit Regulates Auxin and ABA Signaling in Tomato. Plant Physiol..

[35] Yadav D.K., Shukla D., Tuteja N. (2013). Rice Heterotrimeric G-Protein Alpha Subunit (RGA1): In Silico Analysis of the Gene and Promoter and Its Upregulation under Abiotic Stress. Plant Physiol. Biochem..

[36] Liu X., Tian J., Zhou X., Chen R., Wang L., Zhang C., Zhao J., Fan Y. (2014). Identification and Characterization of Promoters Specifically and Strongly Expressed in Maize Embryos. Plant Biotechnol. J..

[37] Chen H., Zou Y., Shang Y., Lin H., Wang Y., Cai R., Tang X., Zhou J.-M. (2008). Firefly Luciferase Complementation Imaging Assay for Protein-Protein Interactions in Plants. Plant Physiol..

[38] Lu Q., Tang X., Tian G., Wang F., Liu K., Nguyen V., Kohalmi S.E., Keller W.A., Tsang E.W.T., Harada J.J. (2010). Arabidopsis Homolog of the Yeast TREX-2 mRNA Export Complex: Components and Anchoring Nucleoporin. Plant J. Cell Mol. Biol..

[39] Shi Q., Xia Y., Wang Q., Lv K., Yang H., Cui L., Sun Y., Wang X., Tao Q., Song X. (2024). Phytochrome B Interacts with LIGULELESS1 to Control Plant Architecture and Density Tolerance in Maize. Mol. Plant.

[40] Hu B., Jiang Z., Wang W., Qiu Y., Zhang Z., Liu Y., Li A., Gao X., Liu L., Qian Y. (2019). Nitrate-NRT1.1B-SPX4 Cascade Integrates Nitrogen and Phosphorus Signalling Networks in Plants. Nat. Plants.

[41] Wang J., Chen L., Long Y., Si W., Cheng B., Jiang H. (2021). A Novel Heat Shock Transcription Factor (ZmHsf08) Negatively Regulates Salt and Drought Stress Responses in Maize. Int. J. Mol. Sci..

[42] Cao J., Yao D., Lin F., Jiang M. (2014). PEG-Mediated Transient Gene Expression and Silencing System in Maize Mesophyll Protoplasts: A Valuable Tool for Signal Transduction Study in Maize. Acta Physiol. Plant..

[43] Bolger A.M., Lohse M., Usadel B. (2014). Trimmomatic: A Flexible Trimmer for Illumina Sequence Data. Bioinformatics.

[44] Kim D., Langmead B., Salzberg S. (2015). HISAT: A fast spliced aligner with low memory requirements. Nat. Methods.

[45] Jiao Y., Peluso P., Shi J., Liang T., Stitzer M.C., Wang B., Campbell M.S., Stein J.C., Wei X., Chin C.-S. (2017). Improved Maize Reference Genome with Single-Molecule Technologies. Nature.

[46] Pertea M., Pertea G.M., Antonescu C.M., Chang T.-C., Mendell J.T., Salzberg S.L. (2015). StringTie Enables Improved Reconstruction of a Transcriptome from RNA-Seq Reads. Nat. Biotechnol..

[47] Love M.I., Huber W., Anders S. (2014). Moderated estimation of fold change and dispersion for RNA-seq data with DESeq2. Genome Biol..

[48] Thimm O., Bläsing O., Gibon Y., Nagel A., Meyer S., Krüger P., Selbig J., Müller L.A., Rhee S.Y., Stitt M. (2004). Mapman: A User-Driven Tool to Display Genomics Data Sets onto Diagrams of Metabolic Pathways and Other Biological Processes. Plant J..

