# Supplementary material for: GPCR-like Protein ZmCOLD1 Regulate Plant Height in an ABA Manner
Source: Int J Mol Sci. 2024 Nov 1;25(21):11755. doi: 10.3390/ijms252111755 (PMC11546568; doi:10.3390/ijms252111755)
Supplement: Supplementary file 1 [file ijms-25-11755-s001.zip › Supplementary Figure.pdf]

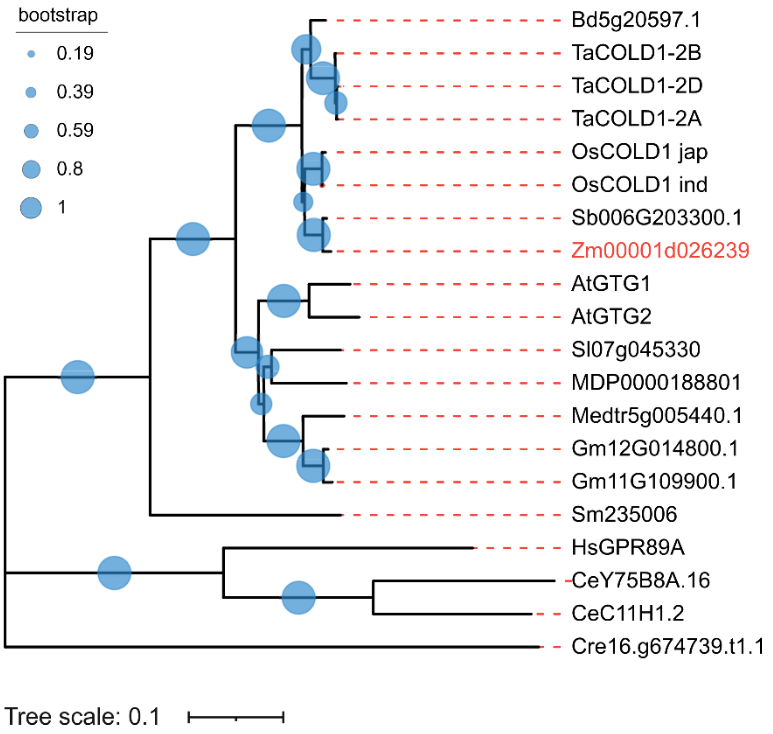

Figure S1 Phylogenetic tree constructed with proteins of ZmCOLD1 and homologs from other species via Neighbor-Joining method. The Gene ID for ZmCOLD1 is in red.

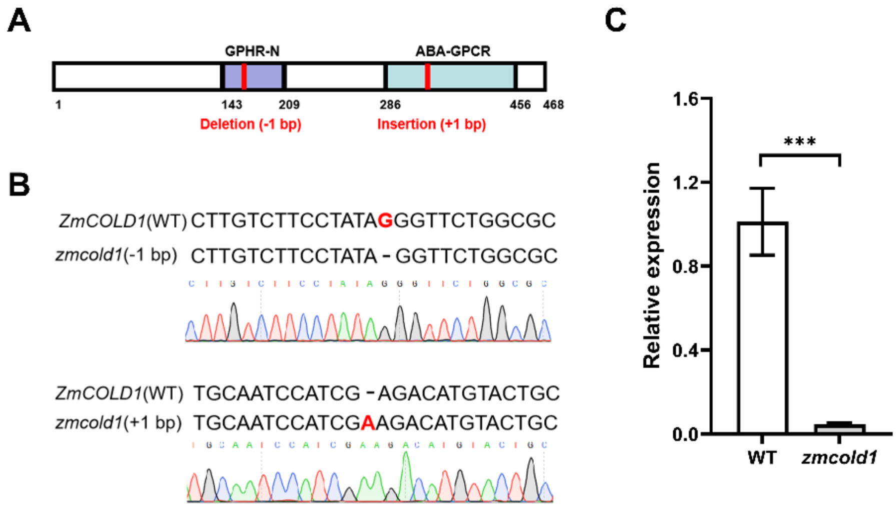

Figure S2 Identification of mutation site and mRNA expression level of *ZmCOLD1* gene in *zmcold1* mutant. (A) Identification of mutation site of *zmcold1* knockout lines. The mutant had a C-base deletion at nucleotide site 471 and an A-base insertion at nucleotide site 969 on the *ZmCOLD1* gene. (B) Sequencing result of mutation site. (C) The relative expression level of *ZmCOLD1* gene expression in *zmcold1* mutant. Expression levels were normalized to that of *ZmACTIN*. Data are mean ± s.d. (n = 9 biologically independent samples). WT, wild type. Asterisks indicate a significant difference compared to those of untreated controls by Student's t test, *** denotes *P*-value < 0.001.

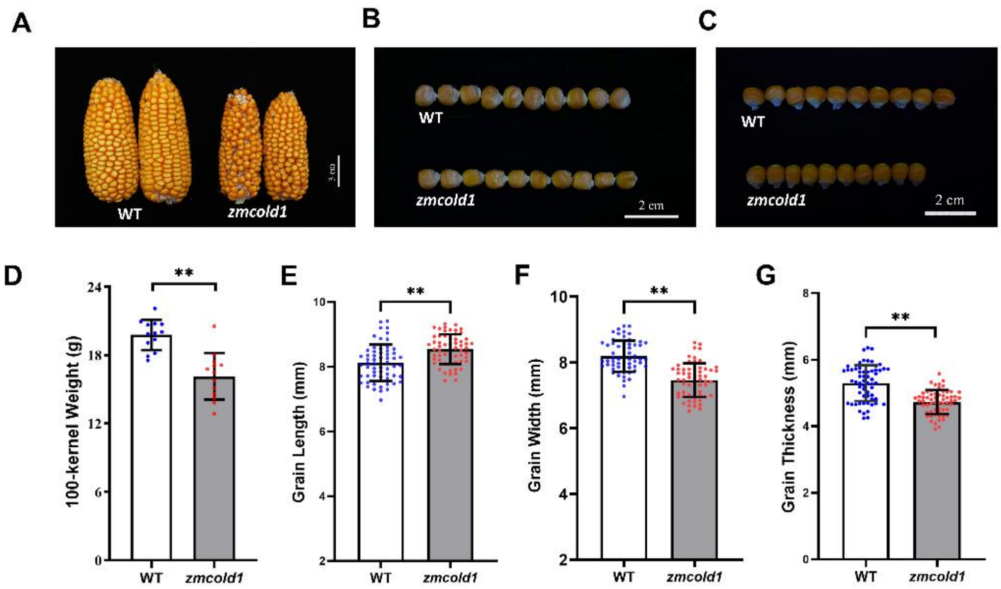

Figure S3 Phenotypes of ear and agronomic traits of grain for *zmcold1* (June 2021, Hefei Anhui). (A): Wild-type and *zmcold1* fruit spike, Scale bars 3 cm. (B) and (C): Seed of wild type and *zmcold1*, (n=10 biologically independent samples), Scale bars 2 cm. (D): The 100-kernel weight of WT and *zmcold1* seeds. Data are mean ± s.d. (n=13 biologically independent samples). (E): Grain length of WT and *zmcold1*. Data are mean ± s.d. (n=60 biologically independent samples). (F): Grain width of WT and *zmcold1*. Data are mean ± s.d. (n=60 biologically independent samples). (G): Grain thickness of WT and *zmcold1*. Data are mean ± s.d. (n=60 biologically independent samples). Asterisks indicate a significant difference compared to those of untreated controls by Student's t test, ** *P*-value<0.01.

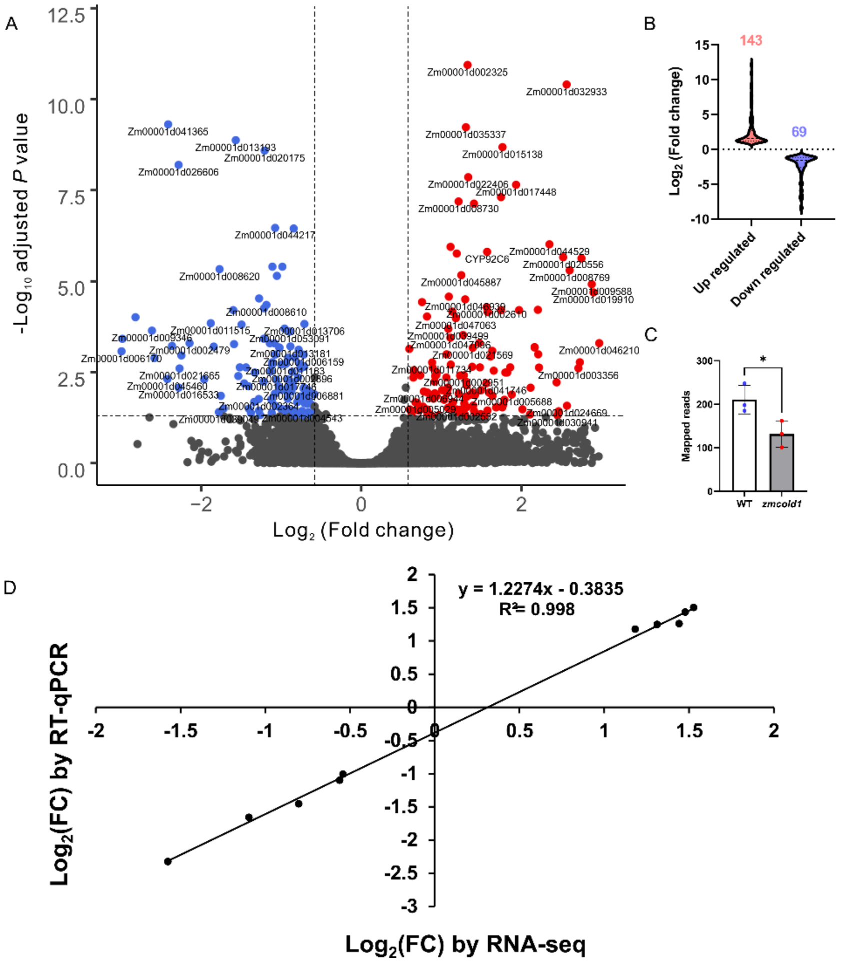

Figure S4. Differentially expressed genes between wild type and mutant. (A) Volcano map of differentially expressed genes. (B) DEGs detected between *zmcold1* and WT plants. (C) Expression of *ZmCOLD1*. * denotes *P*-value < 0.05.（D） Verification of RNA-Seq results by RT-qPCR.
